# The Clinical Frailty Scale (CFS) employment in the frailty assessment of patients suffering from Non-Communicable Diseases (NCDs): A systematic review

**DOI:** 10.3389/fmed.2022.967952

**Published:** 2022-08-16

**Authors:** Nicolò Granata, Martina Vigoré, Andrea Steccanella, Luca Ranucci, Simona Sarzi Braga, Paola Baiardi, Antonia Pierobon

**Affiliations:** ^1^Department of Cardiac Respiratory Rehabilitation of Tradate Institute, Istituti Clinici Scientifici Maugeri IRCCS, Varese, Italy; ^2^Istituti Clinici Scientifici Maugeri IRCCS, Psychology Unit of Montescano Institute, Montescano, Italy; ^3^Cardio-vascular Department, MultiMedica IRCCS, Sesto San Giovanni, Italy; ^4^Central Scientific Direction, Istituti Clinici Scientifici Maugeri IRCCS, Pavia, Italy

**Keywords:** frailty, Clinical Frailty Scale, Non-Communicable Diseases, chronic diseases, systematic review

## Abstract

**Background:**

The Clinical Frailty Scale (CFS) is a well-established tool that has been widely employed to assess patients' frailty status and to predict clinical outcomes in the acute phase of a disease, but more information is needed to define the implications that this tool have when dealing with Non-Communicable Diseases (NCDs).

**Methods:**

An electronic literature search was performed on PubMed, Scopus, EMBASE, Web of Science, and EBSCO databases to identify studies employing the CFS to assess frailty in patients with NCDs.

**Findings:**

After database searching, article suitability evaluation, and studies' quality assessment, 43 studies were included in the systematic review. Researches were conducted mostly in Japan (37.5%), and half of the studies were focused on cardiovascular diseases (46.42%), followed by cancer (25.00%), and diabetes (10.71%). Simplicity (39.29%), efficacy (37.5%), and rapidity (16.07%) were the CFS characteristics mostly appreciated by the authors of the studies. The CFS-related results indicated that its scores were associated with patients' clinical outcomes (33.92%), with the presence of the disease (12.5%) and, with clinical decision making (10.71%). Furthermore, CFS resulted as a predictor of life expectancy in 23 studies (41.07%), clinical outcomes in 12 studies (21.43%), and hospital admissions/readmissions in 6 studies (10.71%).

**Discussion:**

CFS was found to be a well-established and useful tool to assess frailty in NCDs, too. It resulted to be related to the most important disease-related clinical characteristics and, thus, it should be always considered as an important step in the multidisciplinary evaluation of frail and chronic patients.

**Systematic review registration:**

https://www.crd.york.ac.uk/PROSPERO/display_record.asp? PROSPERO 2021, ID: CRD42021224214.

## Introduction

It is well known that one of the most compelling challenges of our time is population aging (1). In recent years, as to the World Health Organization report on aging (1), the number of people aged 65 years or over is progressively increased: it is estimated that for the year 2050 the population over 60 years old will double, reaching almost 22% of the total one. In parallel, the number of people aged 80 years or over is growing even faster, and it is expected to triple by 2050 (2). Aging is often associated with chronicity and multimorbidity and their prevalence increases in people aged 65 years and older (3, 4).

Elderly people often are affected by Non-Communicable Diseases (NCDs), also defined as chronic diseases. It is estimated that each year NCDs are responsible for 71% of all deaths (5). The NCDs can be clustered into four main categories: Cardiovascular, Chronic respiratory diseases, Cancer, and Diabetes. Cardiovascular diseases are responsible for most NCDs deaths, followed by cancers, respiratory diseases, and diabetes (5). Furthermore, old age and chronicity are often associated with frailty syndrome.

Despite the importance and the interest toward frailty, there is no agreement on the definition (6). In fact, according to the literature, two theoretical paradigms try to define frailty: the biomedical and bio-psycho-social paradigms. As to the biomedical paradigm, frailty is considered a biological syndrome in which there is an important reduction in the functional reserves and a diminished resistance to stressors. These features result in a cumulative impairment of the multiple physiological systems that cause a state of increased vulnerability and adverse consequences (7). Conversely, the bio-psycho-social paradigm defines frailty as a dynamic state that affects an individual that loses one or more functional domains (physical, psychological, and social) due to the influence of different variables that increase the risk of adverse health outcomes (8). Despite the differences between the two considered paradigms, it is possible to underline a common conclusion: frailty is associated with the loss of different functional domains, which leads to an increased vulnerability to adverse events such as risk of falls, hospitalization, disability, and mortality (9). Anyhow, it is universally recognized that frailty is a clinical condition that can impair several areas (e.g., general health and operative risk) (10) and, according to the criteria established by Fried, its prevalence is around 10% in ≥65 and between 25–50% in over 85 years old (11). Moreover, in a recent systematic review and meta-analysis, the prevalence data collected from 62 countries and territories showed that the pooled prevalence in studies using physical frailty measures was 12% (95% CI = 11–13%; *n* = 178), compared with 24% (95% CI = 22–26%; *n* = 71) for the deficit accumulation model (those using the Frailty Index, FI) (12).

The overall result of the interaction between the aging process and clinical conditions is the progressive deterioration of the homeostatic balance, so it follows that a deteriorated homeostasis may result in an increased difficulty in coping with stressors (10). People affected by frailty syndrome are more susceptible to health status changes following a minor stress event than non-frail people.

Rockwood et al. proposed an operational definition of frailty with the Frailty Index (FI), by counting the number of deficits accumulated over time, within an extensive list (13, 14). This definition was based on the idea that frailty is a state of chaotic disorganization of physiological systems that can be estimated by evaluating certain indexes such as functional status, diseases, physical and cognitive deficits, psychosocial risk factors, and geriatric syndromes. Furthermore, in 2005, Rockwood et al. described a different approach in frailty evaluation, which was embedded in the Clinical Frailty Scale (CFS), a screening tool based on clinical judgment (14).

CFS, originally developed in Canada, is entirely based on clinical judgment, fast and easy to use, and it has proven to be an effective instrument for frailty assessment (1= Very Fit; 2= Well; 3= Managing Well; 4= Vulnerable; 5= Mildly Frail; 6= Moderately Frail; 7= Severely frail; 8= Very severely Frail; 9= Terminally Ill) (13–16).

According to the scientific literature, the use of CFS in frailty assessment has been widely used to predict patients' outcomes in the acute phase of the disease (16–18). Few studies tried to understand the impact of frailty on rehabilitation outcomes, for example, Holland and colleagues (18) by focusing on pulmonary rehabilitation and Pandey and colleagues (17) on heart failure patients.

More information is needed to define the implications of frailty syndrome, not only in the acute phase of a disease but also in the presence of chronic disease, therefore, this systematic review aims to evaluate the use of CFS for frailty assessment, with a specific focus on chronic and non-communicable diseases.

## Methods

The systematic review was registered on the PROSPERO database that was previously searched for similar reviews in order to avoid duplication: “The Clinical Frailty Scale (CFS) employment in the frailty assessment of patients suffering from Non-Communicable Diseases (NCDs): a systematic review” (PROSPERO 2021 CRD42021224214).

Data were reported according to the international PRISMA (Preferred Reporting Items for Systematic Reviews and Meta-Analyses) guidelines (19) and, a meta-analysis was not conducted due to the wide heterogeneity of the methodologies (20, 21) adopted by the studies considered, so we have conducted a narrative synthesis.

### Search strategy

An electronic literature search was performed on PubMedMedline through Pubmed, Scopus, EMBASE, Web of Science, and EBSCO databases, considering all the publications until December 2021, to describe how CFS is employed with patients suffering from chronic conditions (Appendix a). Different combinations of keywords, including *Clinical Frailty Scale, noncommunicable (or non-communicable) disease/s, chronic disease/s, heart disease/s, cardiovascular disease/s, heart failure, coronary heart disease, hypertension, stroke, cancer, diabetes, chronic obstructive pulmonary disease (or COPD), chronic respiratory disease/s, chronic lung disease/s*, and *asthma*, were entered and applied in the title and abstract sections.

A support from the Microsoft Office^TM^ pack was used: after the Comma-Separated Values (CSV) files were downloaded from the online databases, the organization functions of Microsoft Excel were used to unify all the results in a single sheet and to remove all the duplicated records.

After the electronic search was completed, two reviewers (AS, LR) independently performed the screening of the records retrieved and subsequently, after a full text analysis, they identified the eligible papers. Doubts and concerns about inclusion and exclusion criteria were discussed by all researchers through a triangulation process (NG, MV, AS, LR, AP).

### Inclusion and exclusion criteria

Studies were considered suitable for inclusion if written in English, published in peer-reviewed journals, and where the CFS was employed to screen patients' frailty. There were no limits concerning patients' age, sample size, type of disease/s, and settings where the studies were performed.

Articles that did not deal with frailty, meeting abstract, books/book chapters, comment/editorial, protocol/design, reviews, and meta-analysis, were excluded.

### Data extraction

Information collected through the full-text analysis was extracted by two independent reviewers (NG, MV) and it was organized in a synoptic table, according to the following categories: [A] Characteristics of the study: first author, year of study, nation of the study, nation ranking according to the Human Development Index (HDI) (22), study design, study setting, and professional figures involved; [B] Characteristics of the participants: sample size, mean age, and type of disease/s; [C] CFS-related characteristics: reason/s for CFS utilization, time at which CFS was used (e.g., during outpatient or inpatient visits, retrospectively based on clinical records), study authors' comment on CFS, and CFS related results; [D] Other eventual frailty indexes employed and other eventual outcomes considered (e.g., clinical, functional, and psychological outcomes).

The quality of each study was assessed by two independent reviewers (NG, MV) with the Newcastle Ottawa Scale (NOS) (23). In particular, two adapted versions were used, one for cohort and case-control studies, and one for cross-sectional studies. Using these scales, each study was judged on eight or ten items, categorized into three groups: the selection of the study groups, the comparability of the groups, and frailty-related outcomes. As to cohort and case-control studies, stars are awarded for each quality item, and the highest quality studies are awarded up to nine stars. A study is considered of good quality if there are 3 or 4 stars in the selection domain AND 1 or 2 stars in the comparability domain AND 2 or 3 stars in the outcome/exposure domain. Concerning cross-sectional studies, each study is judged on a 10-point scale and divided into four groups: very good studies (9–10 points), good studies (7–8 points), satisfactory studies (5–6 points), and unsatisfactory studies (0–4 points).

## Results

**Figure 1:**
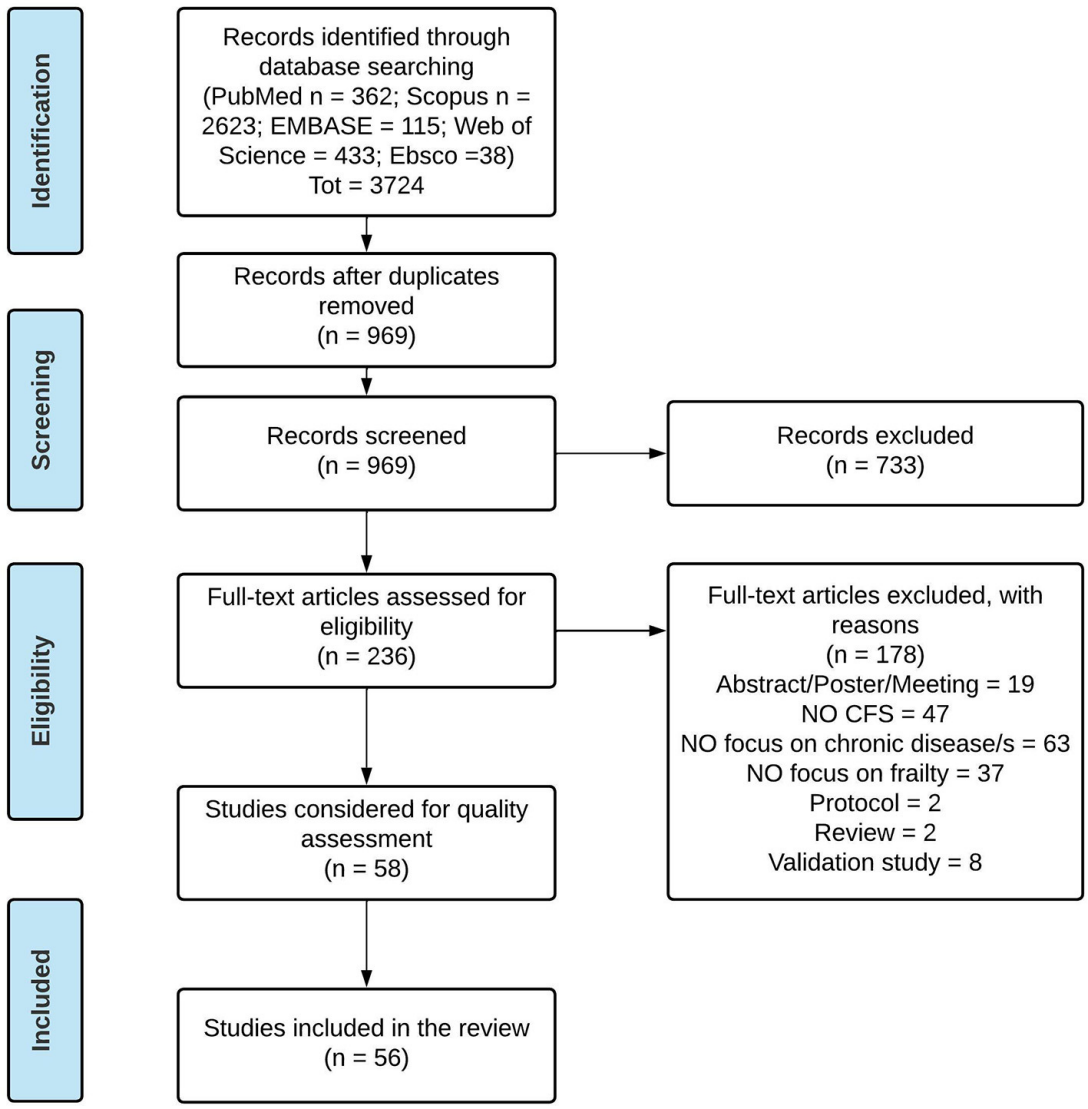
PRISMA Flow Diagram of the systematic review.

After database searching and duplication removal, 969 records were found. Following the title/abstract screening, 236 suitable articles were found and after the full-text reading, 58 studies were considered for quality assessment. Most of the excluded records were not focused on chronic disease/s (*n* = 63), did not include CFS (*n* = 47) or had no focus on CFS (*n* = 37) (Figure 1).

Most studies were of good quality as assessed by the Newcastle-Ottawa Scale (NOS), both for cohort and case-control studies (mean 7.33 ± 0.81), and cross-sectional studies (mean 7.71 ± 0.71). Two articles were excluded as they were judged “fair” quality, particularly in the methodological description of their studies, and they could affect the reliability of the results: for this reason, 56 studies were included and analyzed in the present review.

The information collected is represented in a synoptic table (Appendix a) (24–79). Most of the studies were observational studies (67.86%), followed by retrospective studies (32.14%). The total number of patients in the included studies was 20,497 and the sample sizes varied widely, ranging from 20 patients to 2,588 patients, with most of the studies including more than 100 patients (87.5%) (mean age range: from 42.9 ± 9.4 to 87.4 ± 4.96). Most of the studies were conducted in a hospital (85.71% inpatients and 10.71% outpatients), one study was performed both in inpatient and outpatient settings (1.79%), and only one in a community-dwelling center (1.79%). In all of the studies, CFS was used to assess frailty and for statistical analysis, and in almost half of the studies, it was employed for sample stratification (46.43%) too. In the considered studies, besides CFS, these outcomes were evaluated too: 35.71% functional measures (basic and instrumental activities of daily living, mobility, gait speed, etc.), 23.21% psychological status (anxiety, depression), and 17.86% cognitive functioning. Almost in all of the studies, a physician assessed the CFS score (82.14%), in six cases it was assessed by a nurse (10.71%), in two cases alternatively by a physician or a nurse (3.57%), in one case by an occupational therapist (1.97%), and in one study the patients performed a CFS self-assessment (1.97%). In 10 studies (17.86%), in addition to the CFS, other frailty indexes were employed: the Fried frailty criteria (53, 55, 58, 62), Sarcopenia (44, 45), Frailty index (51, 53), CKD Frailty Index Lab (67), Liver frailty index (55), PARTNER frailty scale (53), Derby frailty scale (51, 52), and Acute frailty (51, 52).

Tables 1a,1b summarize the results concerning the nations of the studies, the type of disease/s, data used for CFS compilation, the CFS evaluation timing, the authors' comment on CFS, and the CFS related results. Tables 1a,1b show that studies were conducted mostly in Japan (37.5%), and almost half of the studies were focused on cardiovascular diseases (46.42%). The other chronic diseases that have been found most frequently were cancer (25.00%) and diabetes (10.71%). In many studies clinical judgement (41.07%), ADL (28.57%), functional capacity status (19.64%), comorbidities (14.29%) and mobility (14.29%) data were used for CFS compilation. The evaluation timing of CFS was: during inpatient clinical visits (19.64%), in the preoperative phase (28.57%), at patients' admission (16.07%), on clinical records of hospitalized patients (10.71%), and retrospectively on clinical records (10.71%). Simplicity (39.29%), efficacy (37.5%), and rapidity (16.07%) were the major authors' comments on CFS. The CFS-related results indicated that CFS was associated with clinical outcomes (33.92%), with the presence of the disease (12.5%), and with clinical decision-making (10.71%). Furthermore, CFS resulted a good predictor of life expectancy (41.07%) and clinical outcomes (21.43%).

**Table 1a:** Main features of the studies included (*n* = 56).

**Nation**	**HDI°(Ranking)**	***n* (%)**	**Disease/s**	***n* (%)**	**Data considered for CFS evaluation**	*n* (%)^*^
Japan	0.919 (19)	21 (37.5)	Cardiovascular disease^a^	26 (46.42)	Clinical judgement	23 (41.07)
UK	0.932 (13)	11 (19.64)	Cancer	14 (25.00)	ADL	16 (28.57)
Canada	0.929 (16)	5 (8.93)	Diabetes	6 (10.71)	Functional capacity status	11 (19.64)
Poland	0.880 (35)	4 (7.14)	Chronic kidney disease	4 (7.14)	Comorbidities	8 (14.29)
Italy	0.892 (29)	3 (5.36)	Cirrhosis	2 (3.57)	Mobility	8 (14.29)
China	0.761 (85)	2 (3.57)	Chronic lung disease	1 (1.79)	Cognitive functions	2 (3.57)
Argentina	0.845 (46)	1 (1.79)	End-stage kidney disease	1 (1.79)	Exhaustion	2 (3.57)
Australia	0.944 (8)	1 (1.79)	End-stage renal disease	1 (1.79)	IADL	2 (3.57)
Germany	0.947 (6)	1 (1.79)			Inactivity	2 (3.57)
Greece	0.888 (32)	1 (1.79)			Preadmission life history	2 (3.57)
Pakistan	0.557 (154)	1 (1.79)			Social support	2 (3.57)
Slovakia	0.860 (39)	1 (1.79)			Symptoms	2 (3.57)
Spain	0.904 (25)	1 (1.79)			Clinical records	1 (1.79)
Sweden	0.945 (7)	1 (1.79)			Comparison to peers	1 (1.79)
Taiwan	n.a. (n.a.)	1 (1.79)			Description of general appearance	1 (1.79)
USA	0.926 (17)	1 (1.79)			Medical examination	1 (1.79)
					Patients' perspective	1 (1.79)
					Psychological distress	1 (1.79)

°HDI index is based on 3 dimensions: a) Life expectance at birth; b) Expected years of schooling and mean years of schooling; c) Gross National Income per capita (United Nations Development Programme, http://hdr.undp.org/en. Accessed on January 2021).

* Refers to the absolute frequency and percentage of each single category retrieved in the included studies (for further details see Appendix b).

a Including: Heart Failure 17.86%, Atrial Fibrillation 8.93%, Aortic Valve Stenosis 7.14%, Coronary Artery Disease 3.57%, Peripheral Artery Disease 3.57%, N-STEMI 1.79%, STEMI 1.79%, Stroke 1.79%.

**Table 1b:** Main features of the studies included (*n* = 56).

**CFS evaluation timing**	n (%)^*^	**Authors'**	*n* (%)^*^	**Study outcomes involving CFS^Δ^**	*n* (%)^*^
		**comment on CFS**			
		**(n=34)^§^**			
Preoperative	16 (28.57)	Simplicity	22 (39.29)	Associated with clinical outcomes	19 (33.92)
Inpatient clinical visits	11 (19.64)	Efficacy (Reliability)	21 (37.5)	Associated with the disease	7 (12.5)
Admission	9 (16.07)	Rapidity	9 (16.07)	Associated with clinical decision making	6 (10.71)
Clinical records of hospitalized patients	6 (10.71)	Subjectivity	4 (7.14)	Associated with socio-demographic characteristics	5 (8.93)
Retrospectively on clinical records	6 (10.71)	Lacking	2 (3.57)	Associated with hospital readmission	1 (1.79)
Outpatient clinical visits	3 (5.36)	Inexpensive	1 (1.72)	Associated with quality of life	1 (1.79)
Discharge	2 (3.57)			Predictor of life expectancy	23 (41.07)
6 months after discharge	1 (1.79)			Predictor of clinical outcomes	12 (21.43)
Home clinical visits	1 (1.79)			Predictor of hospitalization/hospital readmission	6 (10.71)
Initiation of dialysis	1 (1.79)			Predictor of quality of life	1 (1.79)
Postoperative	1 (1.79)			Clinical variables predictors of frailty	2 (3.57)
Not specified	1 (1.79)			Disease predictor of frailty	1 (1.79)
				Socio-demographic characteristics predictors of frailty	1 (1.79)
				CFS not significant	5 (8.93)

* Refers to the absolute frequency and percentage of each single category retrieved in the included studies (for further details see Appendix b).

§ Clustered categories according to authors' comments on CFS (for further details see Appendix b).

Δ Clustered according to the CFS-related results (for further details see Appendix b).

## Discussion

This systematic review was focused on the CFS utilization in patients suffering from chronic diseases (or NCDs), its dissemination in the different nations, the clinical data used to complete it, and the evaluation timing. Moreover, specific attention was dedicated to investigating the CFS characteristics concerning its usability, reliability, and efficacy in predicting disease-related outcomes.

Although CFS is a well-established tool and used worldwide, most of the included studies were conducted in Japan. In a recent scoping review, it was reported that most of the studies were conducted in Canada (80). This inconsistency with the results of the present study could be explained by the specific focus on NCDs, while in the Church and colleagues' review were considered also critical illnesses. Additionally, the elderly population in Japan amounts to more than 30% of the total population (1) and this might explain the dedicated attention to this topic.

In recent years, the number of studies that provided a CFS evaluation is considerably increased, underlining specific attention dedicated to frailty syndrome in different clinical settings and diseases (80). Most of the included studies involved patients affected by chronic cardiovascular diseases (46.42%) and by different types of cancer (25.00%). This prevalence could be due to the impact that these clinical conditions have on mortality since, as highlighted in the WHO report, these diseases account for most of NCDs deaths per year (5).

Frailty is largely considered a geriatric syndrome, but many studies highlight that frailty syndrome has a notable impact on the younger population as well (81). In the present review, five studies (36, 55, 56, 58, 64) considered a sample size of patients with a mean age of <65 years. Although chronic conditions are often associated with the elderly population, scientific evidence shows that NCDs are responsible for 15 million deaths per year in people aged 30–69 years (5). Furthermore, it has been shown that, even though absolute mortality in relation to frailty was higher with increasing age, the relative risk of mortality in relation to frailty was highest for younger people (81). Therefore, efforts to identify, manage, and prevent frailty should include middle-aged individuals with multimorbidity, in whom frailty is significantly associated with mortality, even after adjustment for the number of long-term conditions, sociodemographic characteristics, and lifestyle (82).

All the studies were conducted with an observational or retrospective design. There are no studies in which is described a specific intervention for disease-related frailty and it could be interesting to evaluate the CFS reliability in pre and post-study designs. Indeed, in a recent scoping review, the authors found only few studies conducted in rehabilitation settings (80). Among these, different types and timing of rehabilitation were taken into account, for example, pre-operatory (83, 84) or post-acute rehabilitation (85, 86), and none was focused on NCDs or on chronic diseases. Moreover, a meta-analysis performed by Attwell and Vassallo found only three studies focused on COPD frail patients' rehabilitation (87). This data is consistent with our results since no articles were found about NCDs rehabilitation and it highlights a lack of studies focused on the use of CFS in NCDs rehabilitation. Therefore, specific attention should be given to deepening and shedding light on this topic.

Even though CFS is based on clinical judgment, in six studies (10.71%), the CFS score has been attributed retrospectively based on patients' medical records. This scoring method was reported to be reliable, provided that the charts (medical records, nurse records, etc.,) contain all the elements required to assign a CFS score (88). Also, evidence reports a consistency between CFS scores attributed considering medical records and CFS scores attributed through interviews with patients or their families (89). Moreover, CFS should be administered by medical doctors, but, despite this, ten studies included in this review show that CFS is not always administered by physicians (42, 46, 55–57, 59, 68, 70, 75, 77). This is made possible by the multidimensionality of this tool because it relies on data other than clinical judgment.

In a recent study, the results obtained with the CFS were compared with those obtained with the Edmonton Frail Scale (EFS) (90). The findings of this study imply that the CFS is a valid measurement tool for frailty in critically ill patients, compared with a multidimensional and more comprehensive tool. Similarly, Ritt and colleagues (91), compared this instrument with the Frailty Index, finding that the predictive accuracy of mortality was similar between the two instruments, but the CFS score was even able to predict unplanned hospital admission. Moreover, CFS was found to be an easy-to-use tool and had high inter-rater reliability in addition to a good prognostic value (92). Also in the present review, most of the included studies (82.14%) used only CFS to evaluate frailty. This data is supported by existing literature that reports a high degree of effectiveness of the CFS as a screening instrument (93). Besides, these data may be consistent with this review's results related to the observations on CFS, since most authors commented that it was a simple (Simplicity, 39.29%), reliable (Efficacy, 37.5%), and fast (Rapidity, 16.07%) instrument for frailty assessment.

As for CFS-related results, different studies find associations between CFS score and the disease taken into account, and, conversely, one study finds that the presence of the disease is a predictor of CFS score. These results are supported by the literature, since it was found that chronic diseases contribute to the frailty status development (10) and, in addition, another study suggests a bidirectional association between frailty and the disease, specifically in presence of multimorbidity (94). Same results were found concerning CFS and clinical outcomes: in most of the studies, CFS was found to be associated with or a predictor of patients' clinical outcomes. Literature supports these findings both when it deals with frailty, evaluated with different frailty indexes (95, 96), and when frailty is evaluated specifically with CFS (80). Our results are consistent with the aforementioned studies although they were not focused specifically on chronic diseases.

Moreover, CFS was found to be associated with clinical decision-making, as well. This result is in line with recent literature that outlines the importance of taking into account frailty when dealing with chronic diseases (97, 98). Indeed, frailty is a syndrome that could interact with therapeutic prescriptions for other diseases, worsening the clinical condition, or, on the other side, its course could be accelerated by the implementation of disease-related clinical practices (97, 98).

Socio-demographic characteristics were found to be associated with CFS in different studies. This result is in line with previous literature since frailty is a syndrome that affects particularly older people (99). Moreover, in a recent study, it was found that people with worsening economic conditions over time simultaneously experience a rapid increase in the frailty symptoms (100).

In several articles frailty resulted to be associated with or a predictor of mortality and rehospitalizations. A recent meta-analysis conducted on Chronic Heart Failure (CHF) found that frailty is a significant predictor of all-cause mortality and CHF-related hospitalizations (95). Similar findings are reported in a systematic review on Chronic Kidney Disease and on End-Stage Renal Disease, which suggests that frailty is an independent risk factor of overall mortality in patients affected by these diseases (101). Moreover, Church and colleagues report that several outcomes are associated with CFS score, such as mortality, length of hospitalization, readmissions, and also institutionalizations (80).

Even though different studies focus on the relationship between frailty and quality of life, only one study in this systematic review finds this result. In literature, frailty is associated with worse quality of life in patients affected by different diseases, such as breast and prostate cancer (96, 102), or in cardiovascular diseases (103, 104). Uchmanowicz and colleagues underline that all the areas forming the construct of quality of life (physical, psychological, social, and environmental) are negatively affected by frailty status (103).

In this systematic review, an eventual limitation lies in the labels assigned to group the findings of the included studies, which were created arbitrarily to provide an immediate understanding. Nevertheless, this procedure was conducted by a triangulation process between the reviewers (NG, MV, AS, LR), and supervised by all the authors, to guarantee the best level of objectivity.

On the other side, as far as we know, this is the first systematic review specifically focused on the use of CFS in NCDs, and it could provide useful information both for a further investigation through a meta-analysis and for clinical practice.

## Conclusions

This systematic review provides a specific focus on the utilization of CFS in patients suffering from NCDs that adds useful information in the field of frailty assessment. Indeed, CFS seems to be an easy-to-use and reliable instrument to assess frailty in this kind of disease, it resulted to be associated with a variety of disease-related characteristics, and it is a good predictor of clinical outcomes, life expectancy, hospitalizations, and quality of life. Further research is needed to corroborate these findings, particularly related to CFS predictivity in clinical settings, in order to support a routine assessment of frailty in NCDs patients with this tool. This kind of assessment might be provided also in rehabilitation settings since it provides an overview of patients' frailty status and adds useful information that could be implemented in the tailored rehabilitation program and subsequent intervention.

## Data availability statement

The original contributions presented in the study are included in the article/Supplementary material, further inquiries can be directed to the corresponding author.

## Author contributions

Conceptualization and methodology: NG, MV, SSB, and AP. Investigation: AS and LR. Data curation: NG, MV, AS, and LR. Supervision: SSB and PB. Writing—original draft: NG, MV, AS, and LR. Writing—review and editing: PB and AP. All authors contributed to the article and approved the submitted version.

## Funding

This work was supported by the National Funding (5x1000): Project Name Decadimento cognitivo, fragilità e outcome riabilitativo in pazienti anziani affetti da patologia cardiorespiratoria. DEC_FRAinRIAB (Grant Agreement Number 2424_20 04 20).

## Conflict of interest

The authors declare that the research was conducted in the absence of any commercial or financial relationships that could be construed as a potential conflict of interest.

## Publisher's note

All claims expressed in this article are solely those of the authors and do not necessarily represent those of their affiliated organizations, or those of the publisher, the editors and the reviewers. Any product that may be evaluated in this article, or claim that may be made by its manufacturer, is not guaranteed or endorsed by the publisher.
